# Supplementary figures and images for: Exploring extreme environments in Türkiye for novel P450s through metagenomic analysis
Source: PLoS One. 2025 Sep 8;20(9):e0330523. doi: 10.1371/journal.pone.0330523 (PMC12416667; doi:10.1371/journal.pone.0330523)

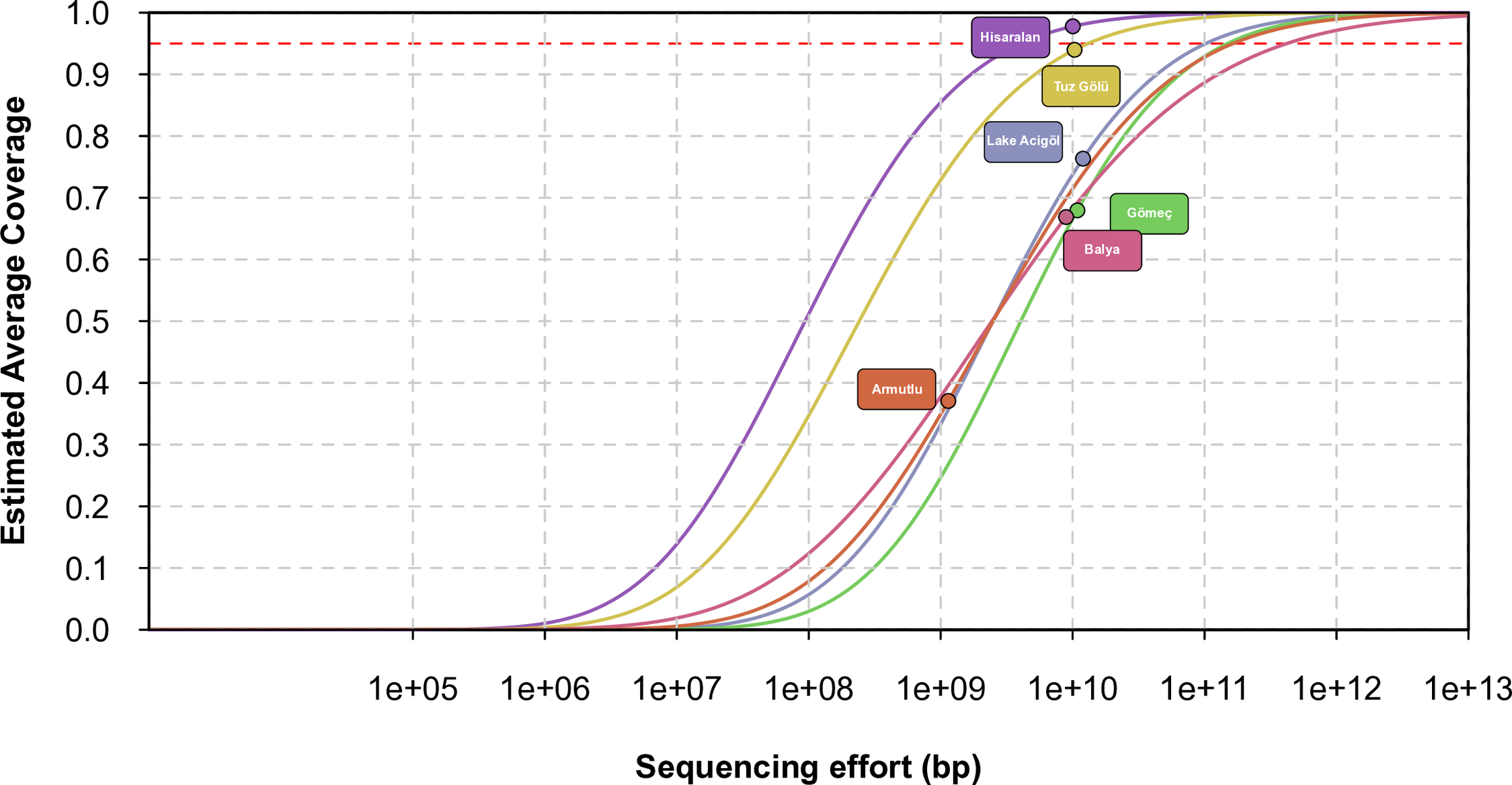

Supplement: S1 Fig — (TIF) [file pone.0330523.s001.tif]

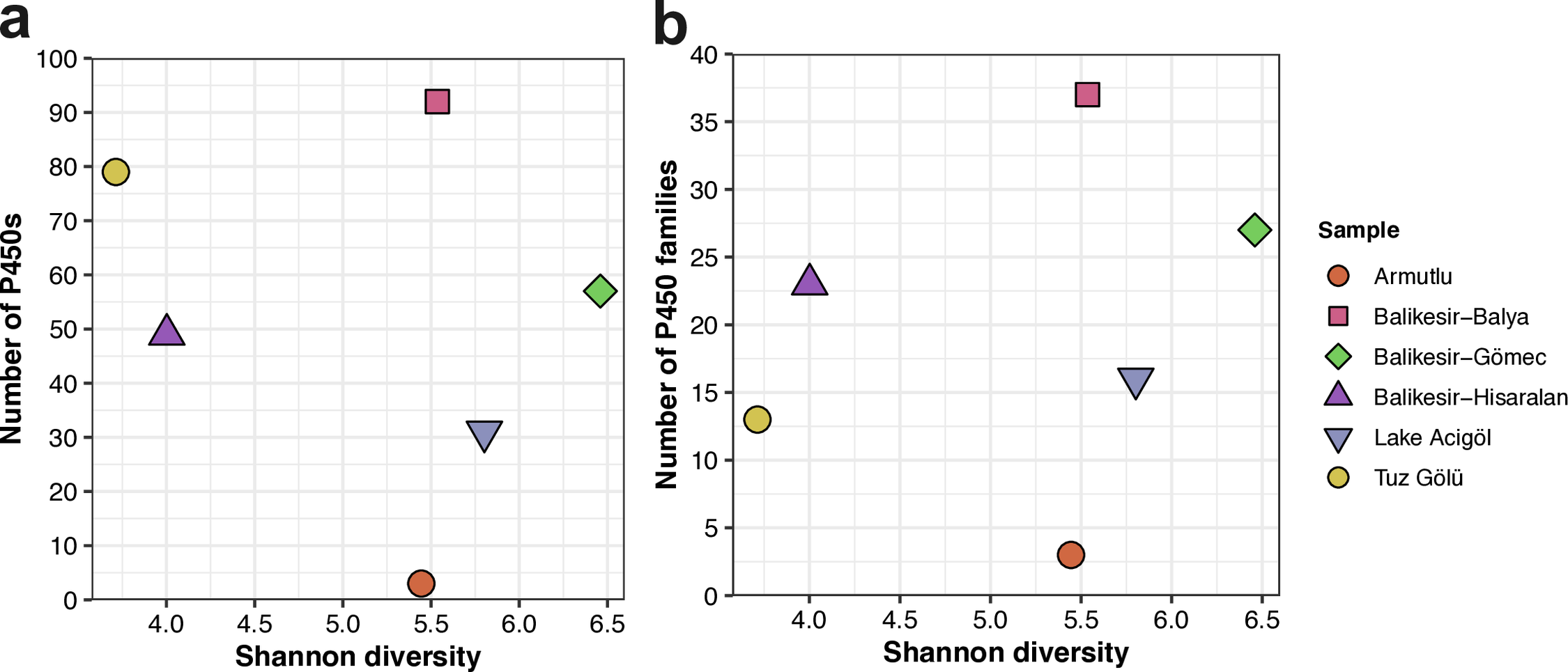

Supplement: S2 Fig — (TIF) [file pone.0330523.s002.tif]

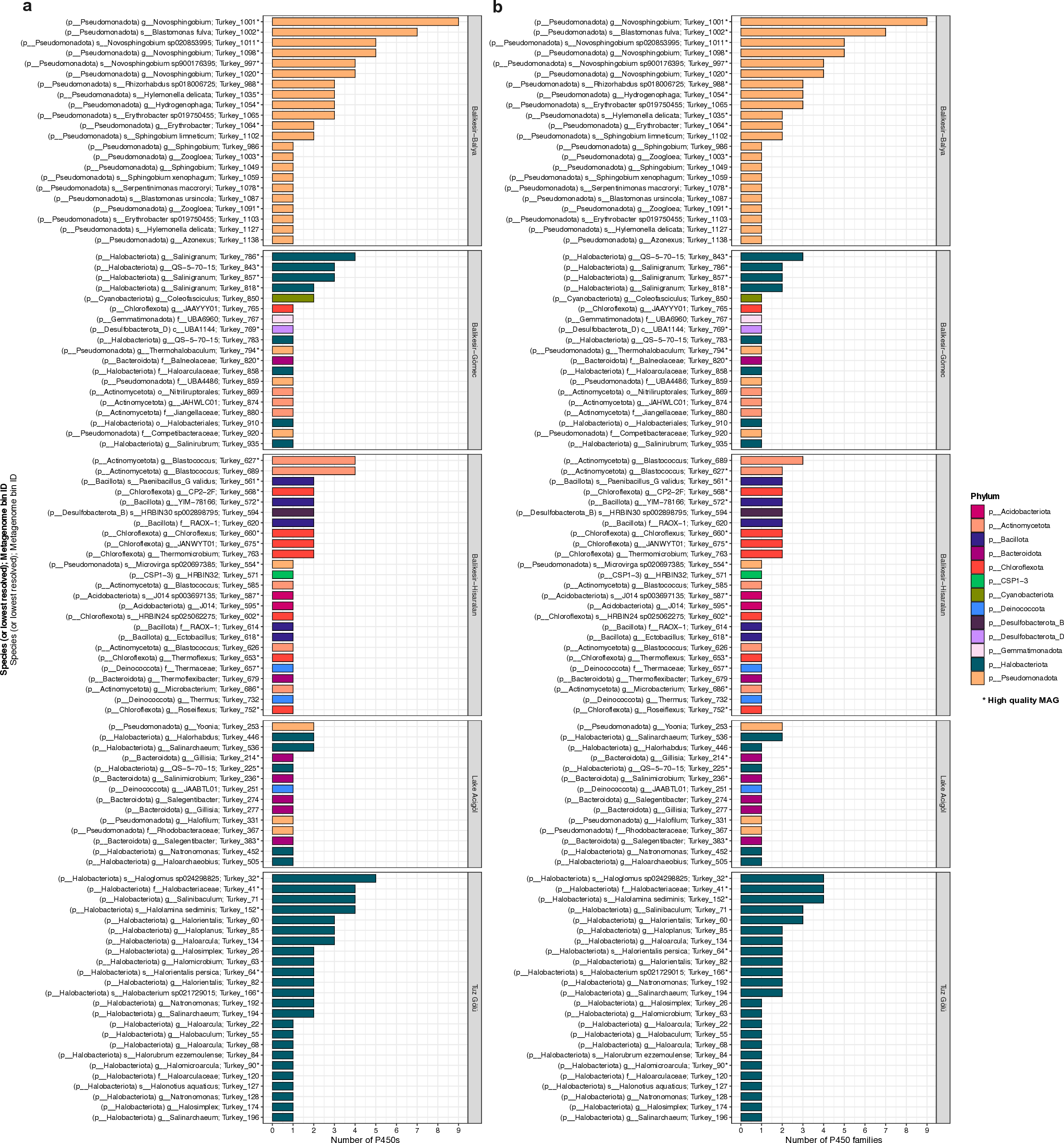

Supplement: S3 Fig — (TIF) [file pone.0330523.s003.tif]
