# Supplementary material for: Exploring extreme environments in Türkiye for novel P450s through metagenomic analysis
Source: PLoS One. 2025 Sep 8;20(9):e0330523. doi: 10.1371/journal.pone.0330523 (PMC12416667; doi:10.1371/journal.pone.0330523)
Supplement: S8 Table — (DOCX) [file pone.0330523.s011.docx]

**Table S8:** **P450s obtained from the six extremophile sample sites and the functions, where known, of previously characterized members of the same P450 family.** Bolded names indicate novel families/subfamilies. Black highlighting indicates novel families. Red highlighting indicates novel subfamilies.

| **Site** | **Extreme Condition** | **P450 Name** | **Function of other, previously characterized members of the same family** |
| --- | --- | --- | --- |
| Lake Acıgöl | Hypersaline | CYP1014G27 | Unknown |
| Lake Acıgöl | Hypersaline | CYP1014G30 |  |
| Lake Acıgöl | Hypersaline | CYP1014J5 |  |
| Lake Acıgöl | Hypersaline | CYP1014L7 |  |
| Lake Acıgöl | Hypersaline | CYP104A15 | Detoxification of plant protective agents [1] |
| Lake Acıgöl | Hypersaline | CYP107PH1 | Synthesis of macrolide antibiotics, biosynthetic pathway of fatty acids, xenobiotics [2] |
| Lake Acıgöl | Hypersaline | CYP107PH2 |  |
| Lake Acıgöl | Hypersaline | CYP107PH3 |  |
| Lake Acıgöl | Hypersaline | **CYP107PJ1** |  |
| Lake Acıgöl | Hypersaline | CYP107PJ2 |  |
| Lake Acıgöl | Hypersaline | CYP109G34 | Fatty acid hydroxylation and oxidation, hydroxylation of testosterone and vitamin D3, monooxygenation of compactin, testosterone and several steroids [3-5] |
| Lake Acıgöl | Hypersaline | CYP1101A32 | Unknown |
| Lake Acıgöl | Hypersaline | CYP1103A20 | Unknown |
| Lake Acıgöl | Hypersaline | CYP1103A21 |  |
| Lake Acıgöl | Hypersaline | CYP1103A22 |  |
| Lake Acıgöl | Hypersaline | CYP1103A23 |  |
| Lake Acıgöl | Hypersaline | CYP1103A24 |  |
| Lake Acıgöl | Hypersaline | CYP1103A25 |  |
| Lake Acıgöl | Hypersaline | CYP1143A3 | Unknown |
| Lake Acıgöl | Hypersaline | CYP152E3 | Decarboxylation and hydroxylation fatty acids (in α- and/or β-position) [6, 7] |
| Lake Acıgöl | Hypersaline | CYP152E4 |  |
| Lake Acıgöl | Hypersaline | CYP153A110 | Hydroxylation of medium- and long-chain alkanes (C5 to C16) [8] |
| Lake Acıgöl | Hypersaline | **CYP1678B1** | Unknown |
| Lake Acıgöl | Hypersaline | CYP174B72 | Terpene metabolism [9] |
| Lake Acıgöl | Hypersaline | CYP1963A17 | Unknown |
| Lake Acıgöl | Hypersaline | CYP1963A18 |  |
| Lake Acıgöl | Hypersaline | **CYP197BC1** | Unknown |
| Lake Acıgöl | Hypersaline | CYP202B26 | Unknown |
| Lake Acıgöl | Hypersaline | CYP202B27 |  |
| Lake Acıgöl | Hypersaline | **CYP2731D1** | Unknown |
| Lake Acıgöl | Hypersaline | **CYP289J1** | Unknown |
| Hisaralan | Hydrothermal | **CYP101Z1** | Hydroxylation of terpenoids [10] |
| Hisaralan | Hydrothermal | CYP107AQ30 | Synthesis of macrolide antibiotics, biosynthetic pathway of fatty acids, xenobiotics [2] |
| Hisaralan | Hydrothermal | CYP107AQ31 |  |
| Hisaralan | Hydrothermal | CYP107AQ32 |  |
| Hisaralan | Hydrothermal | CYP107AZ2 |  |
| Hisaralan | Hydrothermal | CYP107H11 |  |
| Hisaralan | Hydrothermal | CYP107JF5 |  |
| Hisaralan | Hydrothermal | **CYP107PN1** |  |
| Hisaralan | Hydrothermal | CYP109J8 | Fatty acid hydroxylation and oxidation, hydroxylation of testosterone and vitamin D3, monooxygenation of compactin, testosterone and several steroids [3-5] |
| Hisaralan | Hydrothermal | CYP116B304 | Hydroxylation reactions, O-dealkylation,sulfoxidation and epoxidation of a diverse range of compounds (alkylbenzenes, aromatic bicyclic molecules, and terpenoids, polycyclic aromatic compounds) [11, 12] |
| Hisaralan | Hydrothermal | CYP120B16 | Hydroxylation of trans-retinoic acids and converted cis-retinoic acids (9-cis-retinoic acid and 13-cis-retinoic acid), retinal, 3(R)-OH-retinal, retinol, β-apo-13-carotenone (C18) and β-apo-14′-carotenal (C22) [13] |
| Hisaralan | Hydrothermal | CYP123B6 | Hydroxylation of 17β-estradiol [14] |
| Hisaralan | Hydrothermal | **CYP123K1** |  |
| Hisaralan | Hydrothermal | **CYP123L1** |  |
| Hisaralan | Hydrothermal | CYP124B22 | Oxidation of cholesterol derivatives or methyl branched lipids [15] |
| Hisaralan | Hydrothermal | CYP125A80 | Oxidation of cholesterol, 5α cholestane-3β-ol and 4-choles- tene-3-one [16] |
| Hisaralan | Hydrothermal | CYP125A81 |  |
| Hisaralan | Hydrothermal | **CYP125AF1** |  |
| Hisaralan | Hydrothermal | CYP125N2 |  |
| Hisaralan | Hydrothermal | CYP125P3 |  |
| Hisaralan | Hydrothermal | CYP140F2 | Mycolactone synthesis |
| Hisaralan | Hydrothermal | CYP152A25 | Decarboxylation and hydroxylation fatty acids (in α- and/or β-position) [6, 7] |
| Hisaralan | Hydrothermal | **CYP153H1** | Hydroxylation of medium- and long-chain alkanes (C5 to C16) [8] |
| Hisaralan | Hydrothermal | **CYP1681B1** | Unknown |
| Hisaralan | Hydrothermal | CYP1731A5 | Unknown |
| Hisaralan | Hydrothermal | **CYP1731C1** |  |
| Hisaralan | Hydrothermal | CYP1731C2 |  |
| Hisaralan | Hydrothermal | CYP173C5 | Unknown |
| Hisaralan | Hydrothermal | CYP175A3 | Hydroxylation of β-carotene [17] |
| Hisaralan | Hydrothermal | CYP175A4 |  |
| Hisaralan | Hydrothermal | CYP194B15 | Unknown |
| Hisaralan | Hydrothermal | CYP194B16 |  |
| Hisaralan | Hydrothermal | CYP194B17 |  |
| Hisaralan | Hydrothermal | **CYP197AZ1** | Unknown |
| Hisaralan | Hydrothermal | **CYP197BA1** |  |
| Hisaralan | Hydrothermal | **CYP197BB1** |  |
| Hisaralan | Hydrothermal | **CYP197BC1** |  |
| Hisaralan | Hydrothermal | **CYP197BD1** |  |
| Hisaralan | Hydrothermal | CYP197BD2 |  |
| Hisaralan | Hydrothermal | **CYP197Y1** |  |
| Hisaralan | Hydrothermal | CYP239A6 | Unknown |
| Hisaralan | Hydrothermal | CYP253AA2 | Unknown |
| Hisaralan | Hydrothermal | **CYP253AB1** |  |
| Hisaralan | Hydrothermal | CYP268A14 | Hydroxylation of a long chain branched acetate and ketone [18] |
| Hisaralan | Hydrothermal | CYP2745B11 | Unknown |
| Hisaralan | Hydrothermal | CYP2745B12 |  |
| Hisaralan | Hydrothermal | CYP2745B13 |  |
| Hisaralan | Hydrothermal | **CYP2761A1** | Unknown |
| Hisaralan | Hydrothermal | CYP285A10 | Unknown |
| Tuz Gölü | Hypersaline | CYP1001A13 | Unknown |
| Tuz Gölü | Hypersaline | CYP1001A14 |  |
| Tuz Gölü | Hypersaline | CYP1001A15 |  |
| Tuz Gölü | Hypersaline | CYP1002A1 | Unknown |
| Tuz Gölü | Hypersaline | CYP1002A2 |  |
| Tuz Gölü | Hypersaline | CYP1002A8 |  |
| Tuz Gölü | Hypersaline | CYP1002F1 |  |
| Tuz Gölü | Hypersaline | CYP1002G5 |  |
| Tuz Gölü | Hypersaline | CYP1002M10 |  |
| Tuz Gölü | Hypersaline | CYP1002M11 |  |
| Tuz Gölü | Hypersaline | CYP1002M12 |  |
| Tuz Gölü | Hypersaline | CYP1002M13 |  |
| Tuz Gölü | Hypersaline | CYP1002M14 |  |
| Tuz Gölü | Hypersaline | CYP1002M15 |  |
| Tuz Gölü | Hypersaline | CYP1002M9 |  |
| Tuz Gölü | Hypersaline | CYP1002P2 |  |
| Tuz Gölü | Hypersaline | CYP1002P3 |  |
| Tuz Gölü | Hypersaline | CYP1002P4 |  |
| Tuz Gölü | Hypersaline | CYP1002P5 |  |
| Tuz Gölü | Hypersaline | CYP1002Q3 |  |
| Tuz Gölü | Hypersaline | CYP1002S2 |  |
| Tuz Gölü | Hypersaline | **CYP1002Y1** |  |
| Tuz Gölü | Hypersaline | **CYP1002Z1** |  |
| Tuz Gölü | Hypersaline | CYP1003A5 | Unknown |
| Tuz Gölü | Hypersaline | CYP1003B10 |  |
| Tuz Gölü | Hypersaline | CYP1003B9 |  |
| Tuz Gölü | Hypersaline | CYP1014A9 | Unknown |
| Tuz Gölü | Hypersaline | CYP1014G23 |  |
| Tuz Gölü | Hypersaline | CYP1014G31 |  |
| Tuz Gölü | Hypersaline | CYP1014G32 |  |
| Tuz Gölü | Hypersaline | CYP1014G33 |  |
| Tuz Gölü | Hypersaline | CYP1014G34 |  |
| Tuz Gölü | Hypersaline | CYP1014G35 |  |
| Tuz Gölü | Hypersaline | CYP1014G6 |  |
| Tuz Gölü | Hypersaline | **CYP1014M1** |  |
| Tuz Gölü | Hypersaline | **CYP109BL1** | Fatty acid hydroxylation and oxidation, hydroxylation of testosterone and vitamin D3, monooxygenation of compactin, testosterone and several steroids [3, 4] |
| Tuz Gölü | Hypersaline | CYP109G35 |  |
| Tuz Gölü | Hypersaline | CYP109G36 |  |
| Tuz Gölü | Hypersaline | CYP1603A6 | Unknown |
| Tuz Gölü | Hypersaline | CYP174A2 | Terpene metabolism [9] |
| Tuz Gölü | Hypersaline | CYP174A2 |  |
| Tuz Gölü | Hypersaline | CYP174A43 |  |
| Tuz Gölü | Hypersaline | CYP174A44 |  |
| Tuz Gölü | Hypersaline | CYP174A45 |  |
| Tuz Gölü | Hypersaline | CYP174A46 |  |
| Tuz Gölü | Hypersaline | CYP174A47 |  |
| Tuz Gölü | Hypersaline | CYP174A48 |  |
| Tuz Gölü | Hypersaline | CYP174A49 |  |
| Tuz Gölü | Hypersaline | CYP174A50 |  |
| Tuz Gölü | Hypersaline | CYP174A51 |  |
| Tuz Gölü | Hypersaline | CYP174A52 |  |
| Tuz Gölü | Hypersaline | CYP174A53 |  |
| Tuz Gölü | Hypersaline | CYP174A54 |  |
| Tuz Gölü | Hypersaline | CYP174A55 |  |
| Tuz Gölü | Hypersaline | CYP174A56 |  |
| Tuz Gölü | Hypersaline | CYP174A6 |  |
| Tuz Gölü | Hypersaline | CYP174A7 |  |
| Tuz Gölü | Hypersaline | CYP174B11 |  |
| Tuz Gölü | Hypersaline | CYP174B34 |  |
| Tuz Gölü | Hypersaline | CYP174B70 |  |
| Tuz Gölü | Hypersaline | CYP174B71 |  |
| Tuz Gölü | Hypersaline | CYP174B73 |  |
| Tuz Gölü | Hypersaline | CYP174B74 |  |
| Tuz Gölü | Hypersaline | CYP174B75 |  |
| Tuz Gölü | Hypersaline | CYP174C8 |  |
| Tuz Gölü | Hypersaline | CYP174C9 |  |
| Tuz Gölü | Hypersaline | CYP174E7 |  |
| Tuz Gölü | Hypersaline | CYP1963A16 | Unknown |
| Tuz Gölü | Hypersaline | CYP197AL2 | Unknown |
| Tuz Gölü | Hypersaline | CYP197C50 |  |
| Tuz Gölü | Hypersaline | CYP197C51 |  |
| Tuz Gölü | Hypersaline | CYP197C52 |  |
| Tuz Gölü | Hypersaline | CYP197C53 |  |
| Tuz Gölü | Hypersaline | CYP2738A8 | Unknown |
| Tuz Gölü | Hypersaline | CYP2756A1 | Unknown |
| Tuz Gölü | Hypersaline | **CYP2763A1** | Unknown |
| Tuz Gölü | Hypersaline | CYP299A19 | Unknown |
| Tuz Gölü | Hypersaline | CYP299A20 |  |
| Tuz Gölü | Hypersaline | CYP299A21 |  |
| Balya | Acidic | CYP101C10 | Hydroxylation of terpenoid [10] |
| Balya | Acidic | CYP101C7 |  |
| Balya | Acidic | CYP101C8 |  |
| Balya | Acidic | CYP101C9 |  |
| Balya | Acidic | CYP101J6 |  |
| Balya | Acidic | **CYP1055C1** | Unknown |
| Balya | Acidic | CYP107DG12 | Synthesis of macrolide antibiotics, biosynthetic pathway of fatty acids, xenobiotics [2] |
| Balya | Acidic | CYP1083A2 | Unknown |
| Balya | Acidic | CYP1083A6 |  |
| Balya | Acidic | CYP1083A7 |  |
| Balya | Acidic | CYP1083A8 |  |
| Balya | Acidic | CYP1083C5a |  |
| Balya | Acidic | CYP1083C5b |  |
| Balya | Acidic | CYP1089A4 | Unknown |
| Balya | Acidic | CYP108A6 | Terpene hydroxylation, oxidation of polycyclic aromatic hydrocarbons, epoxidation, hydroxylation, demethylation, and dehalogenation of low molecular weight PAHs and their products [19, 20] |
| Balya | Acidic | CYP108A7 |  |
| Balya | Acidic | CYP108D11 |  |
| Balya | Acidic | CYP108D12 |  |
| Balya | Acidic | CYP108D13 |  |
| Balya | Acidic | CYP108D14 |  |
| Balya | Acidic | CYP108D15 |  |
| Balya | Acidic | CYP108D16 |  |
| Balya | Acidic | CYP108D17 |  |
| Balya | Acidic | CYP108D9 |  |
| Balya | Acidic | CYP108G24 |  |
| Balya | Acidic | CYP108H3 |  |
| Balya | Acidic | CYP1090C3 | Unknown |
| Balya | Acidic | CYP1093A2 | Unknown |
| Balya | Acidic | CYP1094A6 | Unknown |
| Balya | Acidic | CYP1094B2 |  |
| Balya | Acidic | CYP1097A21 | Unknown |
| Balya | Acidic | CYP1098A6 | Unknown |
| Balya | Acidic | CYP1098A7 |  |
| Balya | Acidic | CYP1098B8 |  |
| Balya | Acidic | CYP1111A8 | Unknown |
| Balya | Acidic | CYP1112A23 | Unknown |
| Balya | Acidic | CYP111A5 | Oxidation of terpenoids [10] |
| Balya | Acidic | CYP111A6 |  |
| Balya | Acidic | CYP111A7 |  |
| Balya | Acidic | CYP111A8 |  |
| Balya | Acidic | CYP116B171 | Hydroxylation reactions, O-dealkylation,sulfoxidation and epoxidation of a diverse range of compounds (alkylbenzenes, aromatic bicyclic molecules, and terpenoids, polycyclic aromatic compounds) [21, 22] |
| Balya | Acidic | CYP116B21 |  |
| Balya | Acidic | CYP1204A5 | Unknown |
| Balya | Acidic | CYP1204A6 |  |
| Balya | Acidic | CYP125J2 | Oxidation of cholesterol, 5α cholestane-3β-ol and 4-choles- tene-3-one [16] |
| Balya | Acidic | **CYP1294C1** | Unknown |
| Balya | Acidic | CYP1337A3 | Unknown |
| Balya | Acidic | CYP1380A3 | Unknown |
| Balya | Acidic | **CYP145M1** | Unknown |
| Balya | Acidic | CYP145M2 |  |
| Balya | Acidic | CYP150B2 | Oxidation of polyaromatic hydrocarbons and terpenoids and ionone derivatives [23] |
| Balya | Acidic | CYP152B1 | Decarboxylation and hydroxylation fatty acids (in α- and/or β-position) [6, 7] |
| Balya | Acidic | CYP153A130 | Hydroxylation of medium- and long-chain alkanes (C5 to C16) [24] |
| Balya | Acidic | CYP153A131 |  |
| Balya | Acidic | CYP153A8 |  |
| Balya | Acidic | CYP153D26 |  |
| Balya | Acidic | CYP153D32 |  |
| Balya | Acidic | CYP153D33 |  |
| Balya | Acidic | CYP153D34 |  |
| Balya | Acidic | CYP153D35 |  |
| Balya | Acidic | CYP153D36 |  |
| Balya | Acidic | CYP153D37 |  |
| Balya | Acidic | **CYP1698B1** | Unknown |
| Balya | Acidic | **CYP1858B1** | Unknown |
| Balya | Acidic | CYP195A39 | Oxidation of salicylic acids and related aromatic compounds [25] |
| Balya | Acidic | CYP196A17 | Unknown |
| Balya | Acidic | CYP196A33 |  |
| Balya | Acidic | CYP196A34 |  |
| Balya | Acidic | CYP196A35 |  |
| Balya | Acidic | CYP196A36 |  |
| Balya | Acidic | CYP196A37 |  |
| Balya | Acidic | CYP199A42 | Demethylation and demethenylation of para-substituted benzoic acid derivatives and oxidation of benzenes, phenols, benzaldehydes, and benzoic acids [26-28] |
| Balya | Acidic | CYP203A12 | Aromatic ring oxidation [29] |
| Balya | Acidic | CYP203A13 |  |
| Balya | Acidic | CYP203A14 |  |
| Balya | Acidic | CYP203A15 |  |
| Balya | Acidic | CYP203A16 |  |
| Balya | Acidic | CYP203A17 |  |
| Balya | Acidic | CYP203A18 |  |
| Balya | Acidic | CYP219A4 | Unknown |
| Balya | Acidic | CYP225A11a | Unknown |
| Balya | Acidic | CYP225A11b |  |
| Balya | Acidic | CYP226A27 | Oxidation of diterpenoids [30] |
| Balya | Acidic | CYP226A28 |  |
| Balya | Acidic | CYP226A29 |  |
| Balya | Acidic | CYP226A30 |  |
| Balya | Acidic | CYP226D3 |  |
| Balya | Acidic | CYP226D4 |  |
| Balya | Acidic | **CYP2766A1** | Unknown |
| Balya | Acidic | **CYP2767A1** | Unknown |
| Balya | Acidic | **CYP278E1** | Unknown |
| Balya | Acidic | **CYP289K1** | Unknown |
| Gömeç | Hypersaline | **CYP1002AA1** | Unknown |
| Gömeç | Hypersaline | CYP1002G6 |  |
| Gömeç | Hypersaline | **CYP1011H1** | Unknown |
| Gömeç | Hypersaline | CYP1014A10 | Unknown |
| Gömeç | Hypersaline | CYP1014A11 |  |
| Gömeç | Hypersaline | CYP1014G28 |  |
| Gömeç | Hypersaline | CYP1014G29 |  |
| Gömeç | Hypersaline | **CYP107PK1** | Synthesis of macrolide antibiotics, biosynthetic pathway of fatty acids, xenobiotics [2] |
| Gömeç | Hypersaline | **CYP107PL1** |  |
| Gömeç | Hypersaline | **CYP107PM1** |  |
| Gömeç | Hypersaline | CYP107PM2 |  |
| Gömeç | Hypersaline | CYP109G37 | Fatty acid hydroxylation and oxidation, hydroxylation of testosterone and vitamin D3, monooxygenation of compactin, testosterone and several steroids [3-5] |
| Gömeç | Hypersaline | CYP1107B5 | Unknown |
| Gömeç | Hypersaline | CYP110B18 | Fatty acid, terpene and flavanoid hydroxylation [31] |
| Gömeç | Hypersaline | CYP110D25 |  |
| Gömeç | Hypersaline | CYP125N3 | Oxidation of cholesterol, 5α cholestane-3β-ol and 4-choles- tene-3-one [16] |
| Gömeç | Hypersaline | **CYP1318G1** | Unknown |
| Gömeç | Hypersaline | **CYP1321G1** | Unknown |
| Gömeç | Hypersaline | **CYP1528B1** | Unknown |
| Gömeç | Hypersaline | **CYP152AT1** | Decarboxylation and hydroxylation fatty acids (in α- and/or β-position) [6, 7] |
| Gömeç | Hypersaline | **CYP152AU1** |  |
| Gömeç | Hypersaline | CYP152E7 |  |
| Gömeç | Hypersaline | **CYP1540B1** | Unknown |
| Gömeç | Hypersaline | CYP1554A4 | Unknown |
| Gömeç | Hypersaline | CYP174A57 | Terpene metabolism [9] |
| Gömeç | Hypersaline | CYP174B76 |  |
| Gömeç | Hypersaline | CYP174B77 |  |
| Gömeç | Hypersaline | CYP174B78 |  |
| Gömeç | Hypersaline | CYP174B79 |  |
| Gömeç | Hypersaline | CYP174B80 |  |
| Gömeç | Hypersaline | CYP174E6 |  |
| Gömeç | Hypersaline | CYP174H2 |  |
| Gömeç | Hypersaline | CYP174H3 |  |
| Gömeç | Hypersaline | CYP174H4 |  |
| Gömeç | Hypersaline | CYP174H5 |  |
| Gömeç | Hypersaline | **CYP180K1** | Phenolic monoterpen production, geosmin production [32] |
| Gömeç | Hypersaline | **CYP1911C1** | Unknown |
| Gömeç | Hypersaline | **CYP197AY1** | Unknown |
| Gömeç | Hypersaline | CYP197AY2 |  |
| Gömeç | Hypersaline | CYP197C54 |  |
| Gömeç | Hypersaline | CYP197C55 |  |
| Gömeç | Hypersaline | CYP197C56 |  |
| Gömeç | Hypersaline | CYP197D2 |  |
| Gömeç | Hypersaline | CYP197L14 |  |
| Gömeç | Hypersaline | CYP203A19 | Aromatic ring oxidation [29] |
| Gömeç | Hypersaline | CYP223G3 | Unknown |
| Gömeç | Hypersaline | **CYP223H1** |  |
| Gömeç | Hypersaline | CYP223H2 |  |
| Gömeç | Hypersaline | **CYP253AC1** | Unknown |
| Gömeç | Hypersaline | **CYP253AD1** |  |
| Gömeç | Hypersaline | **CYP2745F1** | Unknown |
| Gömeç | Hypersaline | **CYP2762A1** | Unknown |
| Gömeç | Hypersaline | **CYP2764A1** | Unknown |
| Gömeç | Hypersaline | **CYP2765A1** | Unknown |
| Gömeç | Hypersaline | CYP289A30 | Unknown |
| Gömeç | Hypersaline | CYP299A22 | Unknown |
| Gömeç | Hypersaline | CYP299A23 |  |
| Armutlu | Hydrothermal | **CYP2759A1** | Unknown |
| Armutlu | Hydrothermal | CYP194B8 | Unknown |
| Armutlu | Hydrothermal | CYP1380C2 | Unknown |

**References**

1. Salam LB, Obayori OS, Ilori MO, Amund OO. Deciphering the cytochrome P450 genes in the microbiome of a chronically polluted soil with history of agricultural activities. Bulletin of the National Research Centre. 2022;46(1). doi: 10.1186/s42269-022-00947-1.

2. Padayachee T, Lamb DC, Nelson DR, Syed K. Structure–Function Analysis of the Biotechnologically Important Cytochrome P450 107 (CYP107) Enzyme Family. Biomolecules. 2023;13(12). doi: 10.3390/biom13121733.

3. Zhang X, Hu Y, Peng W, Gao C, Xing Q, Wang B, et al. Exploring the Potential of Cytochrome P450 CYP109B1 Catalyzed Regio—and Stereoselective Steroid Hydroxylation. Frontiers in Chemistry. 2021;9. doi: 10.3389/fchem.2021.649000.

4. He F, He Y, Zhou J, Gou J, Ma Q, Zhang X, et al. Molecular modification and application of the key enzyme CYP109E1-H in 25-hydroxyvitamin D3 biosynthesis. Biochemical Engineering Journal. 2025;218. doi: 10.1016/j.bej.2025.109667.

5. Putkaradze N, König L, Kattner L, Hutter MC, Bernhardt R. Highly regio- and stereoselective hydroxylation of vitamin D2 by CYP109E1. Biochemical and Biophysical Research Communications. 2020;524(2):295-300. doi: 10.1016/j.bbrc.2020.01.091.

6. Girvan HM, Poddar H, McLean KJ, Nelson DR, Hollywood KA, Levy CW, et al. Structural and catalytic properties of the peroxygenase P450 enzyme CYP152K6 from *Bacillus methanolicus*. *Journal of Inorganic Biochemistry*. 2018;188:18-28. doi: 10.1016/j.jinorgbio.2018.08.002.

7. Pickl M, Kurakin S, Cantú Reinhard FG, Schmid P, Pöcheim A, Winkler CK, et al. Mechanistic Studies of Fatty Acid Activation by CYP152 Peroxygenases Reveal Unexpected Desaturase Activity. ACS Catalysis. 2018;9(1):565-77. doi: 10.1021/acscatal.8b03733.

8. Liang J-L, JiangYang J-H, Nie Y, Wu X-L, Löffler FE. Regulation of the Alkane Hydroxylase CYP153 Gene in a Gram-Positive Alkane-Degrading Bacterium, Dietzia sp. Strain DQ12-45-1b. Applied and Environmental Microbiology. 2016;82(2):608-19. doi: 10.1128/aem.02811-15.

9. Hilberath T, Urlacher VB, Pohl M. Identification and Characterization of Novel Cytochromes P450 from Actinomycetes: Universitäts- und Landesbibliothek der Heinrich-Heine-Universität Düsseldorf; 2021.

10. Bell SG, Dale A, Rees NH, Wong L-L. A cytochrome P450 class I electron transfer system from *Novosphingobium aromaticivorans*. *Applied Microbiology and Biotechnology*. 2009;86(1):163-75. doi: 10.1007/s00253-009-2234-y.

11. Akter J, Lee JHZ, Whelan F, De Voss JJ, Bell SG. Characterisation of the Cytochrome P450 Monooxygenase CYP116B46 from *Tepidiphilus thermophilus* as a Homogentisic Acid Generating Enzyme and its Conversion to a Peroxygenase. *ChemBioChem*. 2025;26(5). doi: 10.1002/cbic.202400880.

12. Donoso RlA, Ruiz D, Gárate‐Castro C, Villegas P, González‐Pastor JE, de Lorenzo V, et al. Identification of a self‐sufficient cytochrome P450 monooxygenase from *Cupriavidus pinatubonensis* JMP134 involved in 2‐hydroxyphenylacetic acid catabolism, via homogentisate pathway. *Microbial Biotechnology*. 2021;14(5):1944-60. doi: 10.1111/1751-7915.13865.

13. Alder A, Bigler P, Werck-Reichhart D, Al-Babili S. *In vitro* characterization of Synechocystis CYP120A1 revealed the first nonanimal retinoic acid hydroxylase. *FEBS J*. 2009;276(19):5416-31. Epub 20090824. doi: 10.1111/j.1742-4658.2009.07224.x. PubMed PMID: 19703230.

14. Ye X, Peng T, Li Y, Huang T, Wang H, Hu Z. Identification of an important function of CYP123: Role in the monooxygenase activity in a novel estradiol degradation pathway in bacteria. J Steroid Biochem Mol Biol. 2022;215:106025. Epub 20211111. doi: 10.1016/j.jsbmb.2021.106025. PubMed PMID: 34775032.

15. Ghith A, Bruning JB, Bell SG. The catalytic activity and structure of the lipid metabolizing CYP124 cytochrome P450 enzyme from *Mycobacterium marinum*. *Arch Biochem Biophys*. 2023;737:109554. Epub 20230225. doi: 10.1016/j.abb.2023.109554. PubMed PMID: 36842492.

16. Ghith A, Bell SG. The oxidation of steroid derivatives by the CYP125A6 and CYP125A7 enzymes from *Mycobacterium marinum*. *The Journal of Steroid Biochemistry and Molecular Biology*. 2023;235. doi: 10.1016/j.jsbmb.2023.106406.

17. Blasco F, Kauffmann I, Schmid RD. CYP175A1 from Thermus thermophilus HB27, the first β-carotene hydroxylase of the P450 superfamily. Applied Microbiology and Biotechnology. 2004;64(5):671-4. doi: 10.1007/s00253-003-1529-7.

18. Child SA, Naumann EF, Bruning JB, Bell SG. Structural and functional characterisation of the cytochrome P450 enzyme CYP268A2 from *Mycobacterium marinum*. *Biochemical Journal*. 2018;475(4):705-22. doi: 10.1042/bcj20170946.

19. Guo C, Wu Z-L. Construction and functional analysis of a whole-cell biocatalyst based on CYP108N7. Enzyme and Microbial Technology. 2017;106:28-34. doi: 10.1016/j.enzmictec.2017.06.016.

20. Luo A, Wu Y-R, Xu Y, Kan J, Qiao J, Liang L, et al. Characterization of a cytochrome P450 monooxygenase capable of high molecular weight PAHs oxidization from Rhodococcus sp. P14. *Process Biochemistry*. 2016;51(12):2127-33. doi: 10.1016/j.procbio.2016.07.024.

21. Kundral S, Beamish H, Giang PD, Salisbury LJ, Nouwens AS, Khare SK, et al. Characterisation of the Self‐Sufficient Cytochrome P450 CYP116B234 From *Rhodococcus globerulus* and Its Suggested Native Role in 2‐Hydroxyphenylacetic Acid Metabolism. *Microbial Biotechnology*. 2025;18(3). doi: 10.1111/1751-7915.70125.

22. Correddu D, Di Nardo G, Gilardi G. Self-Sufficient Class VII Cytochromes P450: From Full-Length Structure to Synthetic Biology Applications. Trends in Biotechnology. 2021;39(11):1184-207. doi: 10.1016/j.tibtech.2021.01.011.

23. Brezna B, Kweon O, Stingley RL, Freeman JP, Khan AA, Polek B, et al. Molecular characterization of cytochrome P450 genes in the polycyclic aromatic hydrocarbon degrading *Mycobacterium vanbaalenii* PYR-1. *Applied Microbiology and Biotechnology*. 2006;71(4):522-32. doi: 10.1007/s00253-005-0190-8.

24. Nie Y, Chi C-Q, Fang H, Liang J-L, Lu S-L, Lai G-L, et al. Diverse alkane hydroxylase genes in microorganisms and environments. Scientific Reports. 2014;4(1). doi: 10.1038/srep04968.

25. Guo D, Xu F, Bell SG, Pang X, Bartlam M, Wong LL. Purification, crystallization and preliminary crystallographic analysis of CYP 195A2, a P450 enzyme from *Rhodopseudomonas palustris*. *Protein Pept Lett*. 2008;15(4):423-6. doi: 10.2174/092986608784246470. PubMed PMID: 18473959.

26. Furuya T, Kino K. Discovery of 2‐Naphthoic Acid Monooxygenases by Genome Mining and their Use as Biocatalysts. ChemSusChem. 2009;2(7):645-9. doi: 10.1002/cssc.200900054.

27. Bell SG, Yang W, Tan ABH, Zhou R, Johnson EOD, Zhang A, et al. The crystal structures of 4-methoxybenzoate bound CYP199A2 and CYP199A4: structural changes on substrate binding and the identification of an anion binding site. Dalton Transactions. 2012;41(28). doi: 10.1039/c2dt30783a.

28. Coleman T, Wong SH, Podgorski MN, Bruning JB, De Voss JJ, Bell SG. Cytochrome P450 CYP199A4 from *Rhodopseudomonas palustris* Catalyzes Heteroatom Dealkylations, Sulfoxidation, and Amide and Cyclic Hemiacetal Formation. *ACS Catalysis*. 2018;8(7):5915-27. doi: 10.1021/acscatal.8b00909.

29. Nguyen KT, Nguyen NL, Milhim M, Nguyen VT, Lai TH, Nguyen HH, et al. Characterization of a thermophilic cytochrome P450 of the CYP203A subfamily from Binh Chau hot spring in Vietnam. FEBS Open Bio. 2021;11(1):124-32. Epub 20201130. doi: 10.1002/2211-5463.13033. PubMed PMID: 33176055; PubMed Central PMCID: PMCPMC7780096.

30. Smith DJ, Patrauchan MA, Florizone C, Eltis LD, Mohn WW. Distinct Roles for Two CYP226 Family Cytochromes P450 in Abietane Diterpenoid Catabolism by *Burkholderia xenovorans* LB400. *Journal of Bacteriology*. 2008;190(5):1575-83. doi: 10.1128/jb.01530-07.

31. Makino T, Otomatsu T, Shindo K, Kitamura E, Sandmann G, Harada H, et al. Biocatalytic synthesis of flavones and hydroxyl-small molecules by recombinant *Escherichia coli* cells expressing the cyanobacterial CYP110E1 gene. *Microbial Cell Factories*. 2012;11(1). doi: 10.1186/1475-2859-11-95.

32. Senate LM, Tjatji MP, Pillay K, Chen W, Zondo NM, Syed PR, et al. Similarities, variations, and evolution of cytochrome P450s in Streptomyces versus Mycobacterium. Sci Rep. 2019;9(1):3962. Epub 20190308. doi: 10.1038/s41598-019-40646-y. PubMed PMID: 30850694; PubMed Central PMCID: PMCPMC6408508.
