# Supplementary material for: Exploring extreme environments in Türkiye for novel P450s through metagenomic analysis
Source: PLoS One. 2025 Sep 8;20(9):e0330523. doi: 10.1371/journal.pone.0330523 (PMC12416667; doi:10.1371/journal.pone.0330523)
Supplement: Attachment — Submitted filename: Response_to_Reviewers.docx [file pone.0330523.s012.docx]

The referees’ comments are presented in ***blue**, our responses in **black**, and newly added or amended manuscript text in **red**.

**Response to Additional Editor Comments**

*Comment 1: It must be specified in the methodology section under a separate section called data records where the data has been deposited such as the NCBI with the required ascension numbers.

A ‘Data records’ section has now been added to the Methods section:

[lines 244–246] The shotgun sequencing read data for the samples described in this study, as well as the 171 higher quality MAGs obtained from them, have been deposited in the NCBI Sequence Read Archive (SRA) and Genbank, respectively, under the bio-project accession PRJNA979897.

*Comment 2: It is unclear if any custom designed codes have been used. In the methods section there must be a section for code availability in which the authors must specify all the custom designed codes that was used. If none was used, this must be specified.

All analyses were performed with published or publicly available software tools, with non-default parameters clearly defined in the manuscript. Appropriate references and/or links to these tools have been provided in the text. A ‘Code availability’ section has been added to the methods to make this explicit.

[lines 249–250] This section confirms that all analyses were performed using published and/or publicly available tools.

*Comment 3: All figures must be rechecked to comply with the PLOS one illustration standards for publication.

The quality of the figures has been improved using the Preflight Analysis and Conversion Engine (PACE) digital diagnostic tool, as recommended by PLOS. We have ensured that all figures now meet the required resolution and clarity standards for publication.

*Comment 4: There must be an overview of methods implemented at the beginning of the methods section in the form of a schematic representation.

We have added a new schematic figure to the paper (Figure 2) to provide an overview of the methods used.

[lines 157–158] An overview of the experimental and computational (see below) methods used to process the samples is provided in Figure 2.

**Journal requirements**

1- We have re-checked the manuscript and supporting files and confirm that they comply with PLOS ONE’s style requirements.

2- We have added the following statement to the cover letter as requested:

The funders had no role in study design, data collection and analysis, decision to publish, or preparation of the manuscript.

3- The Data Availability file was uploaded during the initial submission. We have now also included the Data Availability Statement under a separate heading within the manuscript as follows:

[lines 244–246] The shotgun sequencing read data for the samples described in this study, as well as the 171 higher quality MAGs obtained from them, have been deposited in the NCBI Sequence Read Archive (SRA) and Genbank, respectively, under the bioproject accession PRJNA979897.

4- Figure 1 has been fully recreated. The base map image was exported from the U.S. Geological Survey’s National Map Viewer (https://apps.nationalmap.gov/viewer/), which provides public domain content. Sampling locations, labels, and all visual elements (scale bar, insets, etc.) were manually added by the authors using PowerPoint.

No copyrighted or proprietary imagery has been used in the revised figure, and it is now fully compliant with the CC BY 4.0 license required by PLOS ONE.

[lines 127] USGS National Map Viewer

**Response to reviewers’ comments and suggestions**

**Reviewer #1 Comments**

*Comment 1: The figure images are blurry. Improve.

The quality of the figures has been improved using the Preflight Analysis and Conversion Engine (PACE) digital diagnostic tool, as recommended by PLOS. We have ensured that all figures now meet the required resolution and clarity standards for publication.

*Comment 2: How many samples were collected?

We collected three samples from each region, and this information has now been explicitly stated in the ‘Sampling’ subsection of the Materials and Methods section.

[line 127] …with three samples collected from each site…

*Comment 3: Check the MS for minor typos. See line 219, there should be a full stop before the word "Notably." Also, check the sentence in line 261, its confusing.

Thank you for pointing this out. We have corrected the minor typo by adding a full stop before **“Notably”** on line 219 (now line 262). Additionally, the sentence on line 261 (now lines 296–301) has been revised for clarity.

**[line 261–262] …**and *Pseudomonadota* (Lake Acıgöl, Armutlu, Balya, Gömeç) (Fig 3, Tables S1–S3). Notably, …

**[lines 297–300] An initial screening of the full protein dataset, conducted using a combination of HMM profile searches and alignment with reference sequences from the CYPED database, identified hundreds of putative cytochrome P450 enzymes. The distribution across the six sites was as follows: 55 from Armutlu, 614 from Balya, 801 from Gömeç, 434 from Hisaralan, 579 from Acıgöl, and 541 from Tuz Gölü.**

*Comment 4: State the extreme conditions for the sites in Table 1, just like in Table 2.

We have now included the extreme conditions for each site in Table 1, ensuring consistency with Table 2. The updated version of the table clearly indicates whether each site is hydrothermal, acidic, or hypersaline.

|  | Armutlu | Balya | Gömeç | Hisaralan | Lake Acıgöl | Tuz Gölü |  |
| --- | --- | --- | --- | --- | --- | --- | --- |
| Extreme Condition | Hydrothermal | Acidic | Hypersaline | Hydrothermal | Hypersaline | Hypersaline | |
| No. of P450s | 3 | 92 | 57 | 49 | 31 | 79 |  |
| No. of families | 3 | 37 | 27 | 23 | 16 | 13 |  |
| No. of subfamilies | 3 | 46 | 45 | 50 | 18 | 29 |  |
| Dominant P450 families |  | CYP108 | CYP174 | CYP107&CYP197 | CYP1103 | CYP174 |  |
| P450 diversity percentage (%)* | 100 | 40.2 | 47.4 | 46.9 | 51.6 | 16.5 |  |

*Comment 5: Table 2: Provide references for previously characterized members. Table 2 is too long and should be taken to Supplementary Section.

References for the previously characterized members in **Table 2** have now been added to ensure proper citation and scientific accuracy. Additionally, **Table 2 has been relocated to the Supplementary Section as Table S8 and replaced with a new Table 2 describing just the newly identified families and subfamilies**.

*Comment 6: There is no caption for the conclusion section. If this is the journal's style, then ignore.

While PLOS does not require a separate caption for the conclusion section, we have added a **“Conclusion”** heading for clarity and consistency within the manuscript.

*Comment 7: The conclusion should be concise and limited to the scope of the study. The conclusion should properly align with the stated objective of the work.

The **Conclusion** section was revised to ensure it is more concise and directly aligned with the study’s objectives.

[lines 523–540] Metagenomics is a powerful tool for discovering novel biocatalysts from uncultured microorganisms. Through shotgun metagenomics and computational analyses, this study has identified 311 P450 sequences, including 8 novel families and 49 subfamilies, from diverse extreme environments across Türkiye. Of these sequences, 237 were associated with 138 metagenomic bins or metagenome assembled genomes (MAGs) of prokaryotic extremophiles, many representing taxonomically novel lineages. These findings underscore the untapped microbial diversity in Türkiye’s extreme environments and their potential as rich reservoirs for novel biocatalysts with applications in industrial and environmental biotechnology.

The taxonomic and P450 diversity uncovered in this study contributes to the growing catalogue of reference data for extremophilic microorganisms and their enzymes. These data can support the development of environment-specific microbial or enzymatic markers, aiding the identification of samples from similar geochemical conditions. Previous studies have shown that metagenomic data carry distinctive environmental signatures; for example, they have been used to infer the geographic origin of ancient samples, map the spatial distribution of antimicrobial resistance, and classify environments using machine learning models. By contributing new reference data and uncovering novel P450 lineages, this study provides a valuable resource for future research into the ecological roles and biotechnological potential of extremophile-derived enzymes.

*Comment 8: The authors may revise the introductory section to highlight the significance of metagenomics for environmental forensics in general terms, thereby giving a general background. The current version seems too focused.

See the following: https://doi.org/10.1016/j.mib.2015.05.005,

https://doi.org/10.1007/s11157-019-09501-4,

https://doi.org/10.1007/978-3-319-97922-9_4,

https://doi.org/10.1038/nrg1709, https://doi.org/10.3389/fenvc.2023.1052697, etc.

We thank the reviewer for this suggestion. We have revised the **Introduction** to provide a broader context on the significance of metagenomics, particularly its role in **environmental forensics**, microbial diversity studies, and functional gene discovery.

[lines 55–71] Metagenomics is a culture-independent approach for studying microbial communities by extracting and sequencing genetic material directly from environmental samples (eDNA). Unlike traditional microbiology, which relies on cultivating microbes in the laboratory, metagenomics provides a comprehensive view of entire microbial communities, including previously unculturable species. This approach offers unprecedented insights into microbial diversity, metabolic functions, and ecological interactions, enabling researchers to study microorganisms in their natural habitats without the need for isolation.

Among metagenomic techniques, shotgun metagenomics has emerged as a powerful tool for exploring the functional capacity of microbial communities. By sequencing all genetic material within a sample, this approach enables the identification of novel genes, biosynthetic gene clusters, and entire metabolic pathways. Through the use of the numerous computational tools that have been developed to process such data, it is now possible to reconstruct the genomes of novel microorganisms and functionally annotate their genes, providing researchers with insight into their ecological roles. This approach is instrumental in identifying new enzymes and biomolecules with potential biotechnological applications, including cytochrome P450 enzymes, which play a crucial role in oxidative metabolism across various biological systems.

[lines 96–112] Extreme environments, including hydrothermal vents, polar deserts, hypersaline lakes, acidic mines, and deep-sea sediments, host diverse microbial communities, collectively known as extremophiles. These microbes have evolved unique adaptive strategies to survive the harsh conditions characteristic of such environments, e.g., high temperatures, salinity, pH, and concentrations of heavy metals. Extremozymes—enzymes found in extremophiles—enable survival under these conditions and exhibit remarkable stability and activity, making them highly valuable for biotechnological applications. P450 extremozymes, in particular, have garnered significant attention due to their diverse catalytic capabilities, but relatively few have been identified to date. Extremophilic P450s characterized to date include members of the self-sufficient CYP116 family, as well as the CYP119, CYP154, CYP174, CYP175, and CYP231 families. Jiang et al. identified three moderately halophilic P450 fatty acid decarboxylases—CYP152L1_ortholog, CYP152L7, and CYP152L8—belonging to the CYP152 family. Moreover, Nguyen and colleagues identified 36 potentially thermostable P450s from water samples collected at Binh Chau hot spring in Vung Tau, Vietnam, through metagenome shotgun sequencing. They also discovered a novel moderately alkali-thermophilic P450 from the CYP203 subfamily, which exhibits optimal activity at 50 °C and pH 8.0.

**Reviewer #2 Comments**

*Comment 1: For the abstract,

- Provide a more explicit statement of the research objective and its broader significance to industrial applications.
- It would be helpful to clarify whether the study confirmed any specific enzymatic activities or if the focus was solely on diversity and classification.
- Consider rephrasing the final sentence to reinforce the practical implications of the study, linking enzyme diversity to specific future applications.

We thank the reviewer for this feedback. The abstract has been updated to the following:

[lines 35–52] Cytochrome P450 enzymes (P450s), particularly those of microbial origin, are highly versatile biocatalysts capable of catalyzing a broad range of regio- and stereoselective reactions. P450s derived from extremophiles are of particular interest due to their potential tolerance to high temperature, salinity, and acidity. This study aimed to identify and classify novel microbial P450 enzymes from extreme environments across Türkiye, including hydrothermal springs, hypersaline lakes, and an acid-mine drainage site. The focus of this study was on classifying the sequence diversity of P450 enzymes in these sites. To that end, shotgun metagenomic analysis of six sites, using de novo binning, phylogenetic analysis, and functional gene annotation, was used to discover 311 putative P450 sequences, assigned to 87 families and 158 subfamilies, including 8 novel families and 49 new subfamilies. Of these, 237 were in 138 metagenomic bins, including 45 high-quality metagenome-assembled genomes. The distribution of P450 families varied across sites, reflecting distinct environmental conditions and microbial community compositions. These findings highlight the untapped potential of Türkiye’s extreme habitats as a source of novel biocatalysts. Beyond their industrial relevance, extremophile-derived P450s may also play key roles in enabling microbial adaptation to harsh environmental conditions, through their involvement in stress-responsive metabolic pathways and structurally resilient enzyme forms. This work provides a foundation for future studies into both their biotechnological applications and ecological functions.

*Comment 2: The information in the introduction could be better organized to create a more cohesive narrative that seamlessly leads to the study’s aim. See specific comments below,

- The study's aim is introduced only at the end of the introduction. Consider weaving this aim into the earlier discussion to provide context and create a clear research trajectory.
- Line 50-61: Not sure whether/how this information is relevant to this specific study. The general discussion of P450 enzymes should be structured to create a logical progression from their fundamental properties and significance to the specific objectives of the study.
- Line 79: Provide examples for the numerous biotechnological applications mentioned here.
- Provide the limitations of previous studies and how this research addresses gaps in knowledge, particularly in relation to extremophilic P450s.
- The mention of metagenomics and -omics sciences are relevant, but the description could benefit from more detail about why these methods are particularly effective for uncovering novel P450 enzymes.
- The examples of characterized P450s (e.g., CYP152, CYP203) are helpful, but the connection to the present study could be more explicitly stated. For instance, how do these findings inspire or relate to the current research?

We have revised the Introduction to address these comments as part of our response to Comment 8 from Reviewer 1. We have incorporated the recommendations to provide a broader context and improve clarity. See changes to [lines 55–71] and [lines 96–112] noted above in response to Reviewer 1’s comment 8.

*Comment 3: The labels in Figures 1–6, especially in Figures 2, 3, 5, and 6, are unclear and difficult, or impossible, to read. The formatting of all figures needs to be revised to ensure that the labels and data points are clearly visible and legible.

The quality of the figures has been improved using the Preflight Analysis and Conversion Engine (PACE) digital diagnostic tool, as recommended by PLOS. We have ensured that all figures now meet the required resolution and clarity standards for publication.

*Comment 4:  Lines 211-227: The significance of identifying dominant phyla and their
environmental roles is mentioned briefly, but the implications for the
study's objectives need to be elaborated. For example, discuss how the
microbial community composition influences the diversity of P450 enzymes.

The reviewer is correct that community composition will influence the diversity of P450s. The paper does already comment on the dominant P450 families and the microbial lineages that they were found in, see the ‘Classification of P450s’ section (lines 274–354 in the original submission). We have now expanded on the relationship between the microbial composition and P450 diversity in the ‘Results’ and ‘Discussion’ sections:

[lines 209–211] Shannon diversity was calculated for each sample from genus level mean coverage values from SingleM using phyloseq.

[lines 314–328] We did not observe a clear correlation between microbial diversity and either the number of P450s or the number of P450 families present in the samples (Fig S2). P450s from Balya, which had a relatively high microbial diversity (Shannon diversity of ~5.5), were only encoded by members of the phylum *Pseudomonadota*. Notably, ten of the higher-quality Balya MAGs encoded multiple P450s (from different P450 families; Table S7, Fig S3). This included five members of the genus *Novosphingobium* that each encoded 4–9 P450s, and a *Blastomonas* *fulva* that encoded 7. At the hypersaline sites, Tuz Gölü and Gömeç (diversities of 3.7 and 6.5, respectively), members of the phylum *Halobacteriota*, specifically the families *Haloarculaceae*, *Halobacteriaceae*, *Haloferacaceae*, and *Salinarchaeaceae*, were the primary encoders with 1–5 P450s each. At the other hypersaline site Lake Acıgöl (diversity of 5.8), P450s were primarily encoded by members of the phyla *Bacteroidota*, *Halobacteriota*, and *Pseudomonadota* (1–2 P450s). Among the hydrothermal sites Hisaralan and Armutlu (diversities of 4.0 and 5.4, respectively) bins were only obtained from Hisaralan, and the top encoders with 2–4 P450s included members of the phyla *Actinomycetota*, *Bacillota*, *Chloroflexota*, and *Desulfobacterota*_B.

[lines 409–417] Although no clear correlation was observed between the microbial diversity (Shannon index) and the number of P450s (and P450 families) across the samples, microbial composition did have a strong influence on both the number and diversity of P450 enzymes. Specifically, members of *Pseudomonadota* and *Actinomycetota* contributed a wide variety of P450 families, including CYP108, CYP109, and CYP153, and often encoded multiple P450s in their respective genomes. In the hypersaline environments, *Halobacteriota* species often encoded CYP174s, while *Bacillota* and *Chloroflexota* were linked to CYP125 and CYP197 diversity in hydrothermal habitats. These results suggest that specific microbial groups may shape the diversity of P450s in extreme environments more than overall microbial diversity.

*Comment 5: Provide a short summary/conclusion statement for first two the results sections: “Taxonomic profiling of the extreme sites, Metagenome assembled genomes (MAGs)”.

We have added concluding sentences to both these sections:

[lines 269–271] The taxonomic composition of these extreme environments reveals a diverse range of archaeal and bacterial lineages, with site-specific differences that may be shaped by distinct selective pressures.

[lines 289–291] The recovered MAGs expand our understanding of the genomic diversity in these extreme environments, revealing several novel lineages that warrant further characterization.

*Comment 6: Revise the sentence in lines 302-303 “Notably, for 64 % of P450 families identified here no family member has yet been characterized functionally” for clarity.

This section has been revised and the percentage recalculated according to the additional edits noted at the end of this response to reviews.

[lines 350–351] Notably, 54% of the P450 families identified in this study have not been previously characterized.

*Comment 7: Revise the title of Table 2 for improved clarity: "Table 2. Classified P450s and the functions were known of previously characterized members of the same family obtained from the six extremophile sample sites”.

Table 2 has been moved to the supplementary file as Table S8, and the title revised to improve clarity.

[Table S8 title] P450s obtained from the six extremophile sample sites and the functions, where known, of previously characterized members of the same P450 family.

*Comment 8: Where possible, references and the source organisms for the known functions that are provided in Table 2.

References have been added as suggested. The relevant information can now be found in the updated Table S8.

*Comment 9: Ensure that figure captions are not embedded within the main text. Instead, place each caption directly below its corresponding figure for better organization and readability.

The figure captions have been formatted according to **PLOS ONE guidelines**, ensuring that they are descriptive, numbered sequentially as cited in the text, and listed at the end of the main manuscript file. Additionally, all figures have been provided as separate high-resolution files in the required format.

*Comment 10: Lines 357-359: The sentence is overly general and lacks specific details. Restructure the sentence providing specific examples, context, and evidence for "various industrial sectors" and "useful activities

This section has been revised.

[lines 400–408] Extreme environments can support diverse, extremophilic microbial communities that have developed unique adaptive strategies to survive, and that can encode enzymes with novel structural and functional properties. Among such enzymes are cytochromes P450, a highly diverse superfamily of enzymes capable of catalyzing a broad range of reactions, including aromatic and aliphatic hydroxylation, heteroatom oxidation, epoxidation, and dealkylation at N-, O- and S-centres. The ability of P450s to transform structurally diverse compounds such as fatty acids, steroids, terpenes, and aromatic hydrocarbons makes them key players in microbial metabolism. The products of these reactions are of value to industry, including in the pharmaceutical, bioremediation, and fine chemical sectors.

*Comment 11: In lines 369–370, the sentence “Additionally, other CYP107 forms are involved in the biosynthesis of other natural products of use in medicine” requires elaboration. Provide examples of these compounds, their roles, and the medical conditions they are used to treat.

We appreciate the reviewer’s suggestion. Examples and associated medical applications of CYP107 enzymes are already provided in lines 419–436 of the manuscript.

*Comment 12: Lines 413–415 state: "Thus, the characterization of the CYP109 families and the elucidation of the functions of the subclasses of the CYP174 family defined in this study is of interest for future studies." Based on previously published data, speculate on the potential functions and advantages of the CYP109 families and CYP174 family subclasses defined in this study, particularly in the context of extremophilic environments.

This section has been revised.

[lines 471–477] Notably, the CYP109 and CYP174 members identified in this study are from subfamilies that have not been characterized in any detail biochemically. While the known substrate profiles of these families do not directly indicate roles in salt adaptation, their prevalence across the hypersaline microbiomes suggests they may possess structural features enabling function under high-salinity conditions. These observations highlight the need for future functional and structural studies to explore their potential halotolerance and biotechnological relevance.

*Comment 13: Lines 423–424 state: “However, uncertainties remain regarding the other identified subfamilies in current study, namely CYP125N, CYP125P, and the newly discovered CYP125AF.” Clarify the intended meaning of this sentence. Does it refer to gaps in knowledge about the specific functions of these subfamilies, their potential roles in extremophilic environments, or both? Additionally, consider elaborating on the significance of addressing these uncertainties and how future studies might resolve them.

This section has been revised.

**[lines 483–486]** Based on this information, it can be hypothesized that members of the CYP125 family, including the newly identified CYP125N, CYP125P, and CYP125AF subfamilies, may contribute to pathways that facilitate microbial adaptation to high temperatures in hydrothermal environments.

*Comment 14: Lines 424–427 state: “On the other hand, the presence of CYP197 in different bacterial phyla has been associated with secondary metabolism [6, 83]; however, biochemical and functional characterization of this family is lacking.” Clarify the intended meaning of this sentence. Does it imply that while CYP197 has been linked to secondary metabolism, its specific roles or mechanisms remain unknown? Additionally, expand on why uncovering the functions of CYP197 in hydrothermal environments is significant. For example, consider how such discoveries could contribute to understanding extremophile adaptations, potential biotechnological applications, or the production of novel metabolites.

We have clarified the meaning in this section.

[line 486–492] Members of the CYP197 family have been found across various bacterial phyla and are frequently encoded within biosynthetic gene clusters associated with secondary metabolism. While their specific enzymatic functions and underlying catalytic mechanisms remain uncharacterized, their presence in both hydrothermal sites suggests a role in the biosynthesis of heat-stable or stress-responsive metabolites. Functional characterization of these enzymes may uncover novel biocatalysts with potential applications in biotechnology and natural product discovery.

**Reviewer #3:**

*Comment 1: Presentation of Program Details:

In the text, when describing the programs used, both the version and the GitHub link are included in parentheses in several places (e.g., lines 142, 143, 147, 148, 154). Although this provides valuable information, the text would be clearer and easier to read if these details were moved to the bibliography or footnotes.

We thank the reviewer for the suggestion. However, we prefer to keep the version numbers in the main text, as we believe they contribute to the transparency and reproducibility of the study. However, to improve readability, the Github link for each tool has been moved to the corresponding reference.

*Comment 2: Formatting of Table 2:

Table 2 spans 12 pages, which makes it quite lengthy. It would be more user-friendly to compress the table. For example, the information in the "P450 Name" column does not necessarily need to be in separate rows. You could merge some rows and list the names separated by commas, provided the other data in those rows is consistent.

**Table 2 has been relocated to the Supplementary Section as Table S8 and replaced with a new Table 2 describing just the newly identified families and subfamilies**.

*Comment 3: Description of Taxonomic Classification:

The authors classify metagenomic reads into various taxonomic groups. It would be beneficial to include a broader description of this classification approach. For example, references to relevant literature could provide additional context:

1. Wajid, B., et al. "Music of metagenomics—a review of its applications, analysis pipeline, and associated tools [Erratum: February 2022, v. 22 (1); p. 137]." (2022).Taxometer: Improving taxonomic classification of metagenomics contigs
2. Kawulok, J., and Deorowicz, D. "CoMeta: classification of metagenomes using k-mers." PloS one 10.4 (2015): e0121453.
3. Ounit, R., et al. "CLARK: fast and accurate classification of metagenomic and genomic sequences using discriminative k-mers." BMC genomics 16 (2015):1-13.
4. Breitwieser, F. P., et al. "KrakenUniq: confident and fast metagenomics classification using unique k-mer counts." Genome biology 19 (2018): 1-10.

We have added a brief overview of the processing steps used by the microbial community profiling tool (SingleM) to the ‘Metagenome community profiling’ subsection and included references to several other profiling tools.

[lines 194–206] To obtain a broader assessment of the community composition of each sample, the microbial community profiler SingleM (ver. 0.16.0) was used. Taxonomic profiling tools typically rely on databases derived from reference genomes, limiting abundance calculations to known species while missing novel taxa. In contrast, SingleM can identify lineages where no genome exists. Briefly, it achieves this by a) analyzing only those reads which cover highly conserved regions of single copy marker genes, b) clustering these reads de novo into operational taxonomic units (OTUs), independent of existing taxonomies, c) taxonomically classifying OTUs against the Genome Taxonomy Database (GTDB), d) per marker gene, estimating the relative abundance of each taxon based on OTU classifications, and e) calculating a trimmed mean abundance taken across all the marker genes. The bacterial and archaeal community composition of each sample was therefore determined by classifying those raw reads corresponding to 59 single-copy genes using the ‘pipe’ tool from SingleM, based on taxonomies derived from the GTDB R08-RS214.

*Comment 4: Applications of Environmental Metagenomics:

The authors highlight that various extreme environments can harbor organisms that produce new, unique P450 enzymes. It might be worth mentioning that this information could also be used in reverse—for example, to classify an unknown sample into a specific environment.

Relevant studies include:

1. Bozzi, Davide, et al. "Towards predicting the geographical origin of ancient samples with metagenomic data." Scientific Reports 14.1 (2024): 21794.
2. Zhelyazkova, Maya, et al. "Origin sample prediction and spatial modelling of antimicrobial resistance in metagenomic sequencing data." Frontiers in Genetics 12 (2021): 642991.
3. Kawulok, J. et al. "Environmental metagenome classification for constructing a microbiome fingerprint." Biology Direct 14 (2019): 1-23.
4. Anyaso-Samuel, et al. "Metagenomic geolocation prediction using an adaptive ensemble classifier." Frontiers in Genetics 12 (2021): 642282.

We thank the reviewer for this insightful suggestion. We agree that metagenomic datasets can not only be used for novel enzyme discovery but also for **predicting the environmental origin of unknown samples** based on microbial and enzymatic signatures. We have expanded the Conclusion section to reflect this broader application, referencing the suggested studies.

[lines 531–540] The taxonomic and P450 diversity uncovered in this study contributes to the growing catalogue of reference data for extremophilic microorganisms and their enzymes. These data can support the development of environment-specific microbial or enzymatic markers, aiding the identification of samples from similar geochemical conditions. Previous studies have shown that metagenomic data carry distinctive environmental signatures; for example, they have been used to infer the geographic origin of ancient samples, map the spatial distribution of antimicrobial resistance, and classify environments using machine learning models. By contributing new reference data and uncovering novel P450 lineages, this study provides a valuable resource for future research into the ecological roles and biotechnological potential of extremophile-derived enzymes.

**Additional edits**

While reviewing and updating the manuscript to address the reviewer comments, we identified that 6 of the 317 P450 sequences included in the original submission were missing the consensus heme binding motif required in functional P450s. We have, as such, opted to remove these 6 sequences from the manuscript and have updated the text and other corresponding results to reflect this. Specifically, Supplementary figures S5–7, and Table S8 have been updated, and the following text has been added:

[lines 304–309] Those with at least a 20% match to microbial P450s were then examined for the integrity of their heme-binding domains using the NCBI CDD (Conserved Domain Database), and those that did not contain the consensus heme binding motif F(x)_n_G/A(x)_m_CxG were removed (where: x is any amino acid; n is typically 2 but up to 5 in some families e.g. CYP152; and m is typically 3 but up to 6 in some families).

[lines 513–520] Finally, rare exceptions to the F(x)_n_G(x)_m_CxG motif used for filtering sequences have been described previously, where one or more of the specified residues is conservatively substituted. However, the Cys residue is almost universally conserved and generally considered to be required for generating the highly reactive oxidizing species, compound I, involved in monooxygenase activity. Notably, among the sequences excluded based on the heme-binding motif was a CYP102A178 sequence that appeared to encode a plausible P450 sequence, with the exception that the conserved Cys was replaced by a Tyr residue. Further work is underway to characterise both the putative Tyr and Cys forms of this enzyme.
